# Rapid ossification of a giant post-operative occipital pseudomeningocele following posterior fossa surgery

**DOI:** 10.1007/s00381-023-05829-z

**Published:** 2023-01-21

**Authors:** Daniel Lewis, Chitra Sethuraman, Dimitrios Varthalitis

**Affiliations:** 1 Department of Paediatric Neurosurgery, Royal Manchester Children’s Hospital, Manchester, UK; 2 Division of Neuroscience and Experimental Psychology, School of Biological Sciences, Faculty of Biology, Medicine and Health, University of Manchester, Manchester, UK; 3 Department of Paediatric Histopathology, Royal Manchester Children’s Hospital, Manchester Academic Health Science Centre, Manchester, UK

**Keywords:** Pseudomeningocele, Brain tumour, Posterior fossa

## Abstract

Pseudomeningocele formation following posterior fossa surgery is a well-recognised complication, occurring in up to 33% of operated cases in some series. Ossification of a cranial pseudomeningocele is, however, an exceptionally rare event with only three prior reported cases. We present the unique case of a paediatric patient who developed rapid ossification of a giant occipital pseudomeningocele following posterior fossa surgery. An 8-year-old female patient underwent a midline posterior fossa craniotomy for resection of an exophytic brainstem low-grade glioma. Post-surgery, the patient developed pan-ventricular hydrocephalus and a large occipital pseudomeningocele, which initially increased in size despite a successful endoscopic third ventriculostomy (ETV) being performed. At approximately 3 months post-surgery, reduction of the pseudomeningocele was observed with associated prominent ossification of the pseudomeningocele wall on computed tomography (CT) imaging. Surgical excision was subsequently undertaken, and intra-operatively, a large ossified pseudomeningocele was found. Follow-up MRI 1 month later demonstrated almost complete resolution of the pseudomeningocele with an associated reduction in the degree of pan-ventricular ventriculomegaly. This case highlights that ossification of even giant pseudomeningoceles can occur over a time period of just a few months and clinicians should consider ossification whenever a change in size or consistency of a post-operative pseudomeningocele is encountered.

## Introduction

Pseudomeningoceles are extradural collections of cerebrospinal fluid (CSF) and their formation is a recognised complication of posterior fossa surgery, occurring in up to 33% of operated cases [1–5]. They are thought to primarily result from disruption of the dural-arachnoid membrane and CSF extravasation into the postoperative surgical bed [2, 6, 7]. Hydrocephalus has also been suggested, however, as a driver of pseudomeningocele formation following posterior fossa surgery [2, 8, 9] with blood and tissue debris thought to promote continuous meningeal irritation, scarring of the subarachnoid space, obstruction of CSF flow, and pressure-related displacement of CSF through any existing dural defect [2, 8, 9]. Despite their observed high frequency, ossification of a pseudomeningocele is a rare event. Pseudomeningocele ossification following spinal surgery is well reported [10–17], but ossification following cranial surgery is exceptionally rare, with only three prior reported cases in the literature [1, 2, 18]. In this report, we present the case of a paediatric patient who developed a giant occipital ossified pseudomeningocele following posterior fossa surgery. This case is unique in that ossification and associated reduction of the occipital pseudomeningocele occurred rapidly, within 4 months of the initial surgery, and within an included literature review, we discuss hypothesised mechanisms of pseudomeningocele ossification and management of this rare occurrence.

## Case description

**Fig. 1:**
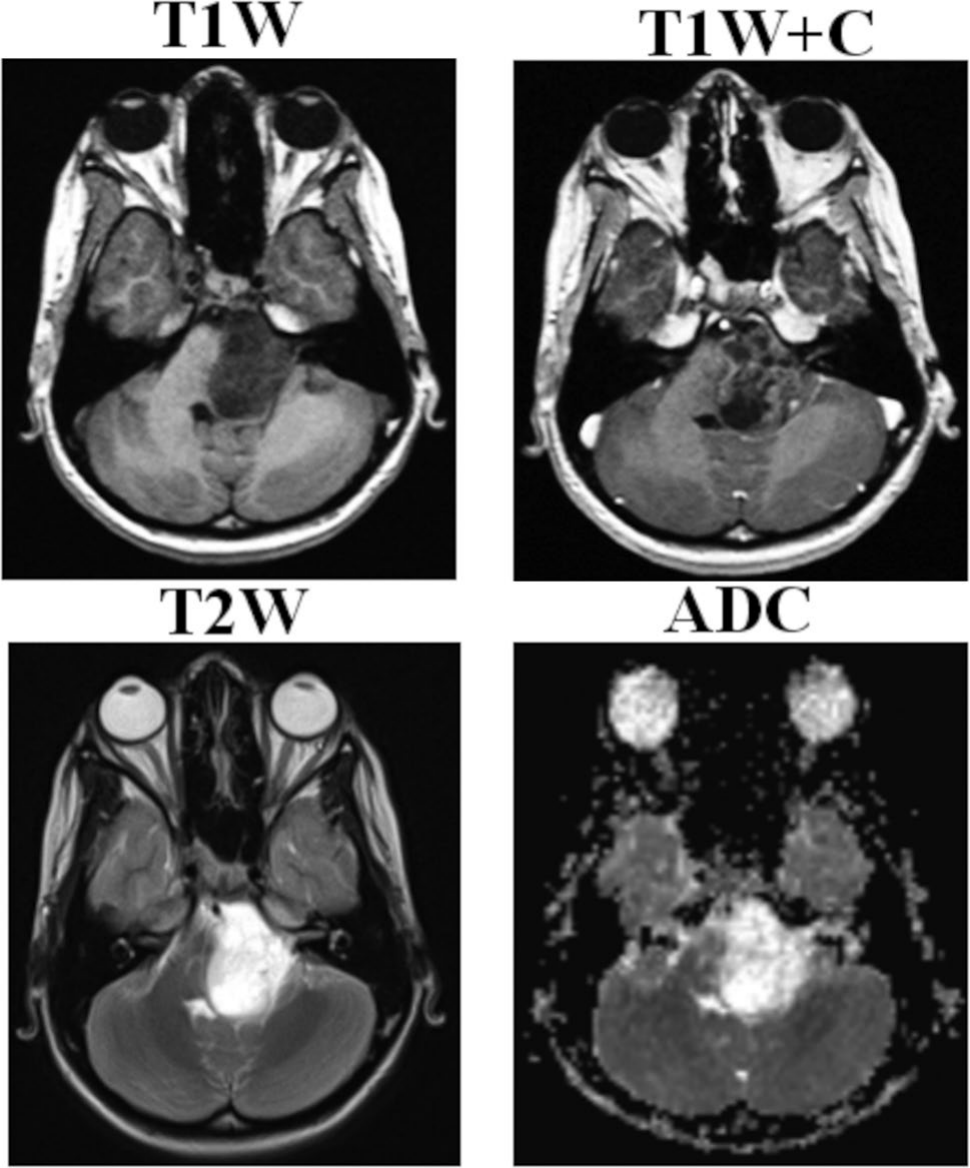
Pre-operative MRI imaging. Selected pre-operative MRI images demonstrating the exophytic tumour arising from the left pons. Note the presence of both solid enhancing components and non-enhancing cystic regions on the post-contrast T1-weighted imaging (T1W + C). Whilst there was some effacement of the IV^th^ ventricle by the tumour, there was no significant pre-operative hydrocephalus observed, and there were no other intracranial or spinal lesions detected. *ADC*, apparent diffusion coefficient; *T1W*, T1-weighted; *T1W* + *C*, post-contrast T1-weighted imaging; *T2W*, T2-weighted

**Fig. 2:**
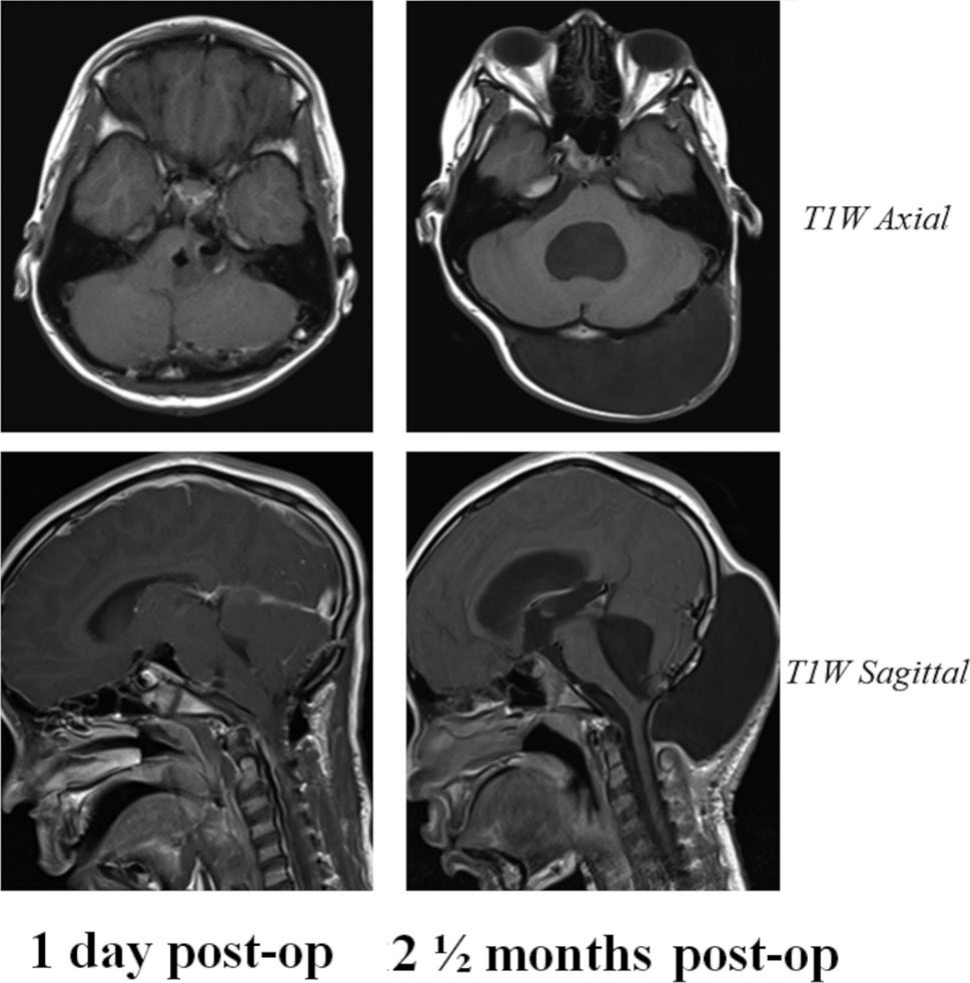
Post-operative MRI imaging showing development of occipital pseudomeningocele. Axial (*top*) and sagittal (*bottom*) T1-weighted images with or without contrast shown. Note at post-operative day 1 (*left panel*) the absence of any significant pseudomeningocele on the pre- or post-contrast T1-weighted imaging. In the weeks following surgery, a large occipital pseudomeningocele developed in association with pan-ventricular hydrocephalus. Despite an ETV attempt with good flow through the III^rd^ ventricular floor stoma (as seen on the sagittal imaging), this pseudomeningocele continued to enlarge up to 2 ½ months post-surgery with persisting ventriculomegaly (*right panel*)

**Fig. 3:**
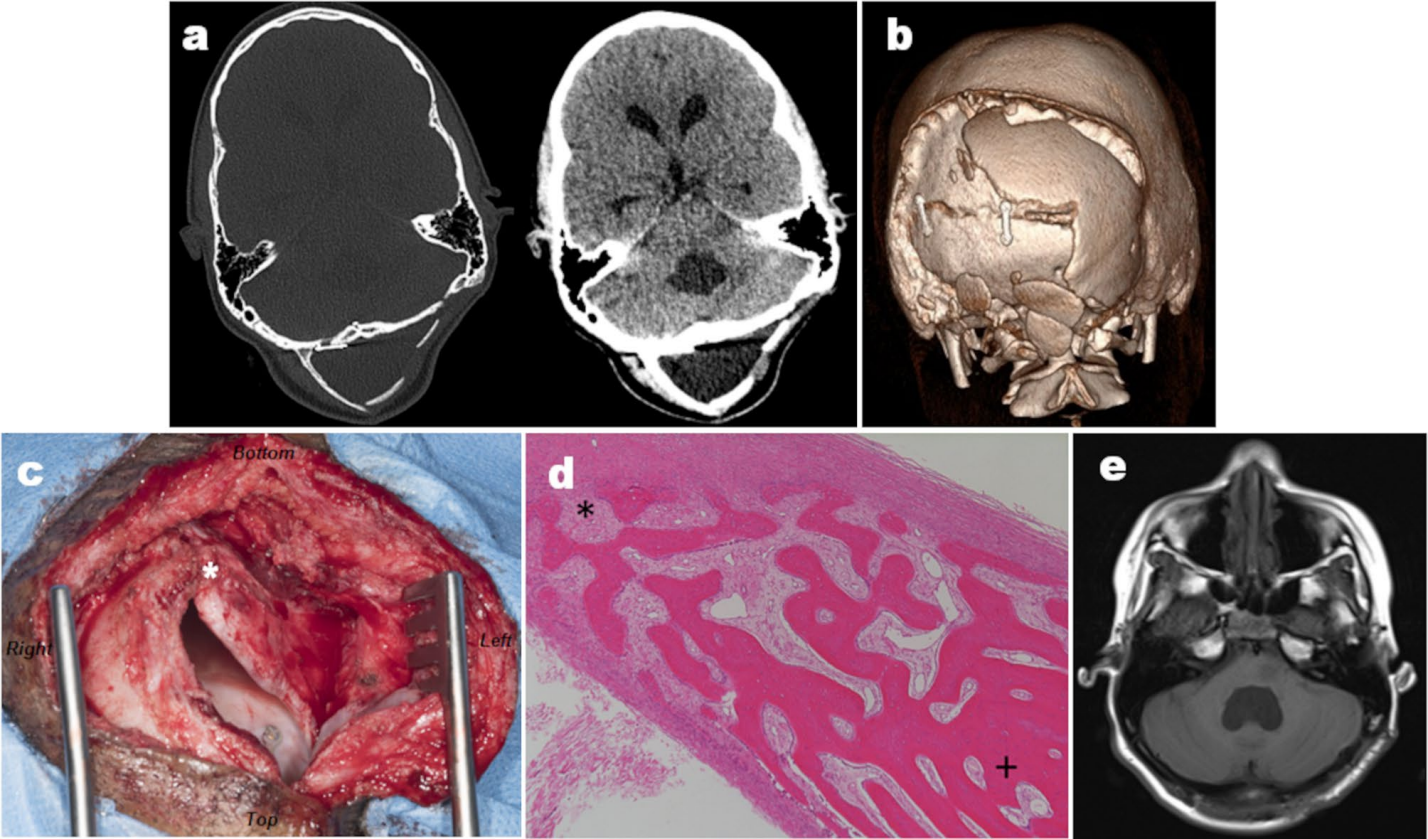
Ossified occipital meningocele. **a** Axial CT imaging performed at 3 ½ months post-surgery demonstrates that there has been an interval reduction in the size of the occipital pseudomeningocele associated with a new rim of ossification around its posterior wall. **b** 3D volume reconstruction of the CT imaging shows this ossification more clearly and its relationship to the underlying bone flap. **c** Intra-operative photograph (looking from cranio-caudal direction) demonstrates the large rim of ossification seen on right posterior pseudomeningocele wall (*). **d** Intra-operative histological specimen (H&E, × 50 magnification) showed thickened fibrous tissue with bony trabeculae. In the peripheral areas, at the interface with fibrous tissue, there was evidence of active bone formation with robust osteoblastic activity (*). Reactive woven bone is identified and towards the centre and there is evidence of remodelling of the lamellae ( +). **e** Axial T1-weighted MRI images taken approximately 1 month after resection of the ossified occipital pseudomeningocele membrane show almost complete resolution of the pseudomeningocele with interval reduction in the degree of pan-ventricular ventriculomegaly

An 8-year-old female presented to our unit with early morning headaches, vomiting, and loss of speech fluency for 1 month. Magnetic resonance imaging (MRI) demonstrated a left pontine exophytic tumour without significant pre-operative hydrocephalus (Fig. 1). The patient subsequently underwent a midline posterior fossa craniotomy with gross total tumour resection, and histology specimens taken intra-operatively confirmed a WHO grade I low-grade glioma. A non-watertight dural closure was achieved at the end of surgery and the bone flap replaced with miniplates and screws, as per standard practice in our institution. On the fourth post-operative day, the patient developed a CSF leak requiring re-suturing and subsequent repeat MRI, obtained due to headaches and vomiting, showed new pan-ventricular hydrocephalus and a large occipital pseudomeningocele (Fig. 2). An endoscopic third ventriculostomy (ETV) was subsequently undertaken with fenestration of both the third ventricular floor and Lillequist’s membrane, with good flow seen across the stoma intra-operatively. Despite symptomatic improvement and good CSF flow across the stoma on post-operative MRI, the pseudomeningocele continued to increase in size over the following 2 ½ months, with an unchanged ventricular configuration (Fig. 2). The patient and her parents were counselled that insertion of ventriculoperitoneal (VP) shunt may be necessary but elected to continue with conservative management and repeat MR imaging at 3 ½ months post-surgery demonstrated reduction of both the pseudomeningocele and the associated ventriculomegaly. Palpable hardening of the pseudomeningocele was observed and subsequent CT imaging demonstrated periosteal reaction around the pseudomeingocele border with new bone formation (Fig. 3a, b). For cosmesis and to preserve skin integrity, surgical excision of this large ossified pseudomeningocele was performed (Fig. 3c), and histological specimens showed fibrous tissue and bony trabeculae with evidence of both active bone formation and bone remodelling in the centre (Fig. 3d). Patient was subsequently discharged from hospital and follow-up MRI taken approximately 1 month after resection of the ossified occipital pseudomeningocele, as part of the usual tumour follow-up, showed almost complete resolution of the pseudomeningocele with an associated reduction in the degree of pan-ventricular ventriculomegaly (Fig. 3e). At last clinic follow-up, the patient continues to make a good recovery from the initial surgery with no symptoms or signs of recurrent extra-cranial pseudomeningocele or hydrocephalus.

## Discussion

To our knowledge, this is only the fourth reported case of ossification of a pseudomeningocele following cranial surgery in the literature, and the first such case following posterior fossa surgery for an intrinsic brain tumour. A unique aspect of this case compared to previous literature reports (Table 1) is the rapidity in which ossification occurred, being evident on CT imaging within 4 months of the initial surgery date.

**Table 1:** Reported cases of ossified cranial pseudomeningocele

**Author**	**Year**	**Age,** **Sex**	**Initial operation**	**Pseudomeningocele location**	**Management**	**Time to ossification after initial surgery**
**Reynolds et al. **[2]	2008	3, Male	Foramen magnum decompression for Chiari type I	Suboccipital	Conservative	Not reported
**Kurzbuch et al. **[1]	2017	3, Female	Foramen magnum decompression for Chiari type I	Suboccipital *In continuity with an intradiploic pseudomeningocele	Excision	6 years
**Bhatt et al. **[18]	2019	12, Male	Ventriculoperitoneal shunt with occipital approach	Occipital	Excision	13 years
**Lewis et al**	2022	8, Female	Midline posterior fossa craniotomy for low-grade glioma	Occipital	Excision	3.5 months

The exact mechanism of pseudomeningocele ossification is debated [1, 2]. Soft tissues surrounding the CSF collection may undergo metaplasia forming cartilage then bone [16, 19] and previous histological reports have demonstrated both fibrous tissue and mature bone in ossified pseudomeningocele [19, 20], a finding similarly demonstrated in our patient case. An interesting observation from this report and previous published cases is that the ossification occurred distant to the craniotomy defect itself, arising from normal calvarial bone and occurring primarily along the posterior pseudomeningocele wall, at the interface with the posterior cervical tissues [2]. It has been hypothesised that residual post-operative haemorrhage at a CSF-soft tissue interface may initiate a local inflammatory reaction and later catalyse pseudomeningocele ossification [2, 20], and the reported presence of haemoglobin and its degradation products within the walls of ossified pseudomeningoceles in previous reports suggests that local haemorrhage may be a prerequisite for ossification [2, 20, 21]. A previous association between Kleeblattschädel/cloverleaf skull deformity (a disorder of premature sutural closure) and ectopic pseudomeningocele ossification has also been reported [2]. No such association was, however, present in our patient, and to our knowledge, there were no known abnormalities of bone formation or metabolism.

In the absence of significant hydrocephalus, most centres will manage postoperative occipital pseudomeningoceles conservatively, waiting at least 7 to 14 days before considering surgical exploration [4, 5]. Early intervention with CSF diversion (ETV or shunting) is recommended though in the presence of hydrocephalus, persistent wound leak, or an enlarging pseudomeningocele [4, 5]. In the present case, an ETV was undertaken prior to consideration of a VP shunt when the pseudomeningocele and ventriculomegaly failed to resolve. Due to patient and parent refusal, VP shunting was never undertaken and an interesting aspect of this case is that following an initial period of demonstrable pseudomeningocele expansion there was spontaneous reduction of the pseudomeningocele and the associated hydrocephalus. Indeed, at the time of the second surgery in this case, no persistent CSF communication between the extra- and intracalvarial compartments was evident.

Although there have been previous reports of spontaneous resolution of giant cranial and spinal pseudomeningoceles [22–24], the exact mechanism behind the spontaneous reduction of the pseudomeningocele in this case is not clear. It can be hypothesised that the same inflammatory- and/or haemorrhage-driven process that resulted in ossification of the pseudomeningocele also resulted in later closure of the dural defect and CSF leak [22, 24]. A reduction in CSF pressure and flow through the dural defect following the ETV, however, also likely played a key role. Although a decrease in the ventricular size was not initially seen following the ETV, a demonstrable flow void was seen on imaging following the procedure [25, 26] and it is recognised that decreases in ventricular size following ETV can be delayed and less evident that those seen following shunting procedures [25–27]. Standard practice following posterior fossa surgery within our unit is to treat patients with hydrocephalus and an enlarging symptomatic pseudomeningocele aggressively with early CSF diversion, through either ETV or shunting. Indeed, this case demonstrates that if steps are taken to achieve CSF pressure normalisation, eventual reduction of enlarging, even giant pseudomeningoceles can occur. As shown in this case, such reduction may also be associated with ossification of the pseudomeningocele wall, and clinicians should consider ossification whenever a change in size or consistency of a post-operative pseudomeningocele is encountered.

## Data Availability

Data sharing not applicable to this article as no datasets were generated or analysed during the current study.

## Abbreviations

ETV: Endoscopic third ventriculostomy
CSF: Cerebrospinal fluid
CT: Computed tomography imaging
MRI: Magnetic resonance imaging
VP: Ventriculoperitoneal
